# Neuronal correlates of cognitive function in patients with childhood cerebellar tumor lesions

**DOI:** 10.1371/journal.pone.0180200

**Published:** 2017-07-10

**Authors:** Johanna L. Reichert, Monika Chocholous, Ulrike Leiss, Thomas Pletschko, Gregor Kasprian, Julia Furtner, Kathrin Kollndorfer, Jacqueline Krajnik, Irene Slavc, Daniela Prayer, Thomas Czech, Veronika Schöpf, Christian Dorfer

**Affiliations:** 1 Institute of Psychology, University of Graz, Graz, Austria; 2 BioTechMed, Graz, Austria; 3 Department of Pediatrics and Adolescent Medicine, Medical University of Vienna, Vienna, Austria; 4 Comprehensive Cancer Center–CNS Tumors Unit (CCC-CNS), Medical University of Vienna, Vienna, Austria; 5 Department of Biomedical Imaging and Image-guided Therapy, Medical University of Vienna, Vienna, Austria; 6 Department of Neurosurgery, Medical University of Vienna, Vienna, Austria; Universidad de Navarra, SPAIN

## Abstract

While it has been shown that cerebellar tumor lesions have an impact on cognitive functions, the extent to which they shape distant neuronal pathways is still largely undescribed. Thus, the present neuroimaging study was designed to investigate different aspects of cognitive function and their neuronal correlates in patients after childhood cerebellar tumor surgery. An alertness task, a working memory task and an incompatibility task were performed by 11 patients after childhood cerebellar tumor surgery and 17 healthy controls. Neuronal correlates as reflected by alterations in functional networks during tasks were assessed using group independent component analysis. We were able to identify eight networks involved during task performance: default mode network, precuneus, anterior salience network, executive control network, visual network, auditory and sensorimotor network and a cerebellar network. For the most ‘basic’ cognitive tasks, a weaker task-modulation of default mode network, left executive control network and the cerebellar network was observed in patients compared to controls. Results for higher-order tasks are in line with a partial restoration of networks responsible for higher-order task execution. Our results provide tentative evidence that the synchronicity of brain activity in patients was at least partially restored in the course of neuroplastic reorganization, particularly for networks related to higher-order cognitive processes. The complex activation patterns underline the importance of testing several cognitive functions to assess the specificity of cognitive deficits and neuronal reorganization processes after brain lesions.

## Introduction

In children, brain tumors are the second most common neoplasms, after leukemia, and constitute 25 percent of all childhood cancers. Tumors of the posterior fossa, including the brain stem and the cerebellum, account for 50 percent of all brain tumors in children [1–3] and comprise pathologies such as medulloblastoma, cerebellar astrocytoma, ependymoma, and brainstem glioma [4].

Due to enormous progress concerning cancer treatment, survival rates of children (ages 0–14 years) with brain and central nervous system tumors have increased significantly in the last decades to a current 5-year relative survival rate of 72.1% [5]. As a result of the increasing number of survivors, treatment-related side effects as well as long-term consequences of childhood brain tumors are well investigated. Reported sequelae of posterior fossa tumor lesions range from motor and visuo-spatial difficulties to disturbances in cognitive and behavioral functions and language [6–10]. Such findings have contributed to a “shift in the understanding of the cerebellum” [11]: While traditionally, the cerebellum was primarily associated with movement coordination, in recent years this view has widened, as clinical, anatomical, and functional studies support the cerebellum’s role for cognitive and emotional functions [6,8,9,11–24].

While cognitive sequelae of cerebellar tumor lesions as assessed by neuropsychological testing are well reported, functional neuroimaging studies are relatively rare. In a recent study by King et al. [25], survivors of childhood posterior fossa tumors were assessed with a working memory task with fMRI. The results showed more prefrontal activation in survivors than in matched controls during the task. As increased activation in these regions was correlated with lower working memory performance, the authors suggested the activation might reflect increased efforts to achieve cognitive control by the patients. The same group also described an increased functional connectivity in frontal regions during resting-state fMRI in a subsample of the patients [26].

Given the frequency and significance of posterior fossa tumors in children, the primary aim of the present study was to assess the impact of cerebellar tumor surgery on different aspects of executive functioning (attention, inhibition, working memory), and on the recruitment of functionally connected higher-order cognitive networks. In contrast to a previous study investigating patients on average 5.3 years after treatment [27], we focus on longer-term effects of tumor lesions, as reorganization processes might occur even years after treatment. Moreover, in contrast to [25,26], to foster sample homogeneity we focus exclusively on patients treated with surgery only, as opposed to treatments including chemotherapy or radiation.

Based on the literature presented before, we expected that patients after cerebellar tumor surgery would show lower performance in neurocognitive tasks than healthy controls. Moreover, we expected a pattern of both increased task-modulation of functional networks (reflecting either compensatory processes or a higher cognitive load) and of decreased task-modulation of networks including cerebellar regions in patients compared to controls. We expected strongest group differences for the tasks with highest cognitive load.

## Materials and methods

The presented study was part of a larger study protocol, which was evaluated and approved by the ethics committee of the Medical University of Vienna (EK No. 370/2011) according to the Declaration of Helsinki.

### Subjects

Data from 17 healthy subjects (6 females, 11 males; *mean* age = 22.40 years; *SD* = 3.3; range: 15–30) and 11 patients after cerebellar tumor surgery (4 females, 7 males; *mean* age = 22.62 years; *SD* = 5.0; range: 14–31) were included. Healthy participants were recruited by public bulletins and were mainly medical students of the Medical University of Vienna. Patients were recruited from a patient database of the Medical University of Vienna. All patients had suffered from a cerebellar low-grade tumor (WHO 1) treated by surgery only. Mean time between tumor surgery and the fMRI measurement was 16.0 years (*SD* = 3.9; range 7.77–20.53 years; see Table 1 for further sample characteristics). Healthy subjects and patients did not differ regarding age (*t*(26) = .14, *p* = 0.887) and gender distribution (*Χ*^2^(1) = .003, *p* = .95). Subjects were informed about the aim of the study and gave their written, informed consent prior to participation. For minors written consent was obtained from a parent or guardian as defined by the local ethics committee.

**Table 1:** Detailed description of patient sample including age, gender, lesion site, and time between surgery and experiment.

ID	Gender	Age	Lesion Site	Time (yrs) between 1^st^ surgery and fMRI measurement
01	m	24.21	vermis	20.53
02	f	23.92	right cerebellum + vermis	20.18
03	m	23.44	right cerebellum	14.04
04	m	21.27	vermis	14.14
05	m	31.25	right cerebellum + vermis	17.56
06	f	16.15	left cerebellum	11.81
07	m	14.94	right cerebellum + vermis	7.77
08	f	18.99	vermis	15.56
09	m	21.71	vermis + right cerebellum	16.33
10	m	22.88	vermis	17.87
11	f	30.08	left cerebellum	20.16

### Procedure and materials

Testing always followed the same study protocol for every participant: The fMRI examination was carried out between 2:30 and 3:00 p.m. Before the scanning session, all participants received task-specific verbal instructions while an instruction screen and example trials were shown. Neuropsychological assessment (see below) was performed after fMRI examination on a separate day; the mean duration between the two examinations was 56 days. All neuropsychological investigations were conducted at the same time of day, 09:30 a.m. In two participants, for organizational reasons the two measurements had to be conducted on the same day, and the neuropsychological testing was carried out before the fMRI examination.

### MRI data acquisition

Functional images were acquired on a 3 Tesla Scanner (Philips Medical System, Best, The Netherlands) using single-shot-gradient-recall echo-planar imaging (EPI). Thirty-five axial slices (4 mm thickness, matrix size of 96x96, FOV of 230 mm, TE/TR of 35/3000 ms) were acquired. Slices were adjusted parallel to the anterior and the posterior commissure and covered from the base of the skull (first section, including the cerebellum) to the cerebrum (as far as possible). Structural MRI scans were performed using a 3D MPRAGE sequence with TR/TE = 2300/4.21 ms, flip angle 90° degrees, inversion time 900 ms and a voxel size of 1x1x1.1 mm^3^, and a field of view of 240x256x176 mm^3^.

The task design for the fMRI investigation was adopted with changes from the tasks “alertness” (AL), “incompatibility” (IC) and “working memory” (WM) from the neuropsychological test battery TAP 2.3 [28], implemented in E-Prime 1.1 software (Psychology Software Tools, Pittsburgh, PA, USA). While performance in the AL task reflects general wakefulness and basic attention capability of participants, IC draws on response inhibition ability, the processing of divergent stimulus information and the ability to suppress interferences in conflict situations. The 2-back WM task requires the ability of short-term storage and of updating information in working memory. In brief, during AL, participants had to press a button each time a cross appeared on the screen. In IC, an arrow appeared on either the left or right side of the screen. Participants had to respond by left/right button press depending on the direction the arrow was pointing, irrespective of the side on which the arrow was appearing. For WM, a sequence of single-digit numbers was shown and participants had to react by button press when the number currently shown corresponded with the last but one number (“2-back task”). For further task description and details of stimulus timing see S1 Fig–S3 Fig and S2 Table. Tasks were implemented in a block design (30 seconds per block), including 6 baseline and 5 active blocks for each paradigm. Experiments were always performed in the order AL, IC, WM except for one subject, caused by technical matters. For WM, a control condition was also presented, during which participants had to press a button each time a pre-defined number was shown (see S4 Fig). Subjects responded by button presses (index finger) on a response board placed on top of their lower body. For AL and WM they always used their right index finger, while for IC, both index fingers were used.

### Neuropsychological assessment

As this substudy was part of a larger project protocol, details of this assessment will be reported elsewhere. For characterization of our study sample, we report IQ scores assessed with the Wechsler Intelligenztest für Erwachsene (WIE, [29]), a German version of the Wechsler Adult Intelligence Scales (WAIS-III, [30]). Of note, two participants (one patient, one control) were 14.9 and 15.8 years old respectively. As the lowest age-norm category for the WIE starts at 16 years of age, we used this category for these two participants.

### Data analysis

#### Behavioral data analysis

Statistical analyses of the behavioral data were performed using the Statistical Package for the Social Sciences, Version 20.0 (SPSS, Chicago, Illinois). The alpha level for all tests was set at *p* = .05 (two-tailed). Reaction times and accuracy of patients and controls were compared using Mann-Whitney tests (including exact significance estimation), as for some parameters, the assumption of normal distribution was not fulfilled. Accuracy was calculated by dividing the number of correct reactions by the total number of trials. For the WM task, the rate of correctly recognized target stimuli (amongst all targets) was analyzed as well (subsequently referred to as ‘hit rate’). For reaction time analyses, the median RT of each individual and task was used to minimize the effect of extreme scores. Due to technical matters, reaction times of the alertness task during the fMRI investigation could not be recorded.

#### MRI data analysis

Since the aim of this study was not only to give insight on the impact of cerebellar tumor surgery on task performance, but also to quantify inter-regional relationships, group independent component analyses (ICA) were conducted to identify functionally connected networks during task performance.

fMRI data were preprocessed using SPM12b (http://www.fil.ion.ucl.ac.uk/spm/software/spm12/) implemented in MATLAB (Matlab 7.14.0, Release 2012a, Mathworks Inc., Sherborn, MA, USA) including slice-time correction, motion correction, spatial normalization to an MNI template, and spatial smoothing with a 8-mm Gaussian kernel (full-width at half-maximum).

Subsequently, group ICA was performed on the pre-processed data of each task [31,32] using the GIFT toolbox, version 4.0a (http://mialab.mrn.org/software/gift/index.html). We chose to conduct separate analyses for each task, as we were interested in task-specific networks and did not want to blur differences between tasks by joining them in one analysis. Group ICA allows model-free analyses of fMRI data and has been widely applied for analysis of both resting-state data and task-based fMRI data. This technique is capable of extracting hidden sources underlying signals and can thus be applied to reveal temporally coherent patterns of blood oxygen level dependent (BOLD) change. As ICA is a data-driven technique, no assumption of the shape of fMRI time courses (a definition of a model) is necessary for identification of functional networks by means of group ICA. We used the Infomax algorithm and 20 runs of the ICASSO toolbox implemented in GIFT to assess algorithmic stability. The “GICA”-algorithm implemented in the GIFT toolbox was applied for back-reconstruction of components. The optimal number of components was estimated for each task by means of a modified minimum description length (MDL) algorithm implemented in the GIFT toolbox. Before ICA, data was further pre-processed by removing image means at each time point. Then, principal component analysis (PCA) was used to reduce data dimensionality. Two data reduction steps were applied: First, data from each subject was reduced to 40 dimensions. This number was chosen at it is recommended to retain more components in the first (subject-level) PCA than in the second (group-level) step [33]. Secondly, the compressed datasets of all subjects were concatenated and then reduced to the number identified by the MDL algorithm (WM: 31, AL: 29, IC: 28 components). The output of the group ICA consists of spatial maps (comprising functionally connected areas) and associated time courses for each component and subject. These can then be used to identify and discard artifactual components (see below) and to apply second-level analyses to determine task-modulation and spatial extent of the remaining components.

Subsequently, components of interest were selected. We followed a three-step procedure to discard artifactual components. In a first step, in accordance with previous recommendations [34], the average power spectra of all components were examined. More specifically, dynamic range (defined as difference between peak power and minimum power at the right side of the peak, see [34]) and the ratio of low frequency (LF) to high frequency (HF) power was assessed for each component by means of the “component viewer” tool of the GIFT toolbox. Low frequencies were defined as < .07 Hz, while high frequencies were defined as .1–.167 Hz. In view of the results reported in [34] [see Figure 3 of the respective article], we discarded components with dynamic range < .031 or LF/HF < 4. In a second step, the remaining component maps were spatially correlated with a cerebral spinal fluid (CSF) map co-registered to the functional images. All components with a spatial correlation of R^2^ > .05 with this map were discarded. In a third step, the remaining components were visually inspected to identify potentially remaining artifactual components. As indices of artifactual components, we regarded e.g. activations on opposite sides of the brain, activations in the ventricles or eyes [35]. For each of the three tasks, the number of components remaining after each step is shown in S1 Table.

Task-relatedness of these components was assessed by multiple regression of the component time courses with the design matrix from SPM12. The design matrix contained the onsets of active task blocks convolved with a hemodynamic response function. The regression resulted in subject-specific beta weights for the task regressor. These beta weights represent the degree to which each component time course was related to the task regressor relative to the (implicit) fixation baseline. The significance of the resulting beta-weights was evaluated by one-sample t-tests for each group, while group differences were assessed with two-sample t-tests. As level of significance for each test, we used a FDR-corrected threshold taking into account the number of components tested. For group comparisons, we additionally report differences significant at p < .05 for components containing cerebellar regions (min. 300 voxels classified as cerebellar by automatic anatomic labeling [36] in FDR-thresholded component maps of the respective task) due to our prior hypothesis of group differences in these areas. When group differences were observed, we conducted correlation analyses between beta-weights and parameters of task performance (accuracy and reaction times). In case of a violation of the assumption of normal distribution, non-parametric Spearman correlations were used.

For visual assessment, the spatial component maps of the components remaining after the component selection procedure were converted to z-scores, with higher z-scores indicating greater contributions of the respective voxel to the component time course [37]. Subsequently, the spatial component maps of each task underwent one-sample t-tests and were thresholded at an FDR-corrected threshold of p_FDR_ < .001 and *k* = 100. This threshold did not influence the statistical analyses and only served to identify and illustrate main regions comprised in the component maps. Masks of the thresholded component maps were created using the xjView toolbox (http://www.alivelearn.net/xjview) and overlaid for visualization using the Mango viewer (http://ric.uthscsa.edu/mango/mango.html).

## Results

### Cognitive assessment

Patients’ *mean* standardized IQ score in the WIE [29] was 97.44 (*SD* = 12.69, range 79–119), while the controls’ *mean* score was 119.02 (*SD* = 10.71, range 100–135). The two groups differed significantly in IQ scores (*t*(26) = -4.85, *p* < .001). Moreover, the scores of both groups were compared to normative mean scores (IQ = 100). This analysis showed that the control group scored significantly higher than the normative mean *(t*(16) = 7.32, *p* < .001), while the patients’ scores were not significantly different from normative mean scores (*p* > .05).

### Behavioral task performance

#### Incompatibility task

Mann Whitney tests showed that neither accuracy nor reaction times differed significantly between patients and controls (*p*s > .05, see Table 2).

**Table 2:** Group comparisons of performance in the three cognitive tasks.

	Accuracy^a^		*Total N*	*U*	*p*	*z*	*r*
Task	Patients	Controls					
Incompatibility							
*Accuracy*	.85 (.04)	.84 (.05)	28	69	.254	-1.163	-.21
*Reaction time*	500.82 (93.11)	452.82 (53.67)	28	64	.172	-1.388	-.26
Working memory							
*Accuracy*	.97 (.02)	.97 (.07)	24	41.5	.051	-1.875	-.38
*Hit rate*	.88 (.21)	.90 (.28)	24	60	.563	-.825	-.17
*Reaction time*	766.95 (164.80)	548.89 (100.26)	24	18	.001	-3.045	-.62
Alertness							
*Accuracy*	1	1	28				

^a^ accuracy values indicate means (standard deviation in parentheses). See section “Behavioral data analysis” for calculation of accuracy and hit rates.

*r* as measure of effect sizes was calculated as *z/√N (see* [38]). Reaction times are reported in milliseconds.

#### Working memory task

One patient and three control participants had to be excluded from this analysis: The patient and one control misunderstood task instructions, one control showed excessive movement artifacts, and one control decided to stop participation.

For WM, there was a tendency of lower accuracy for patients (*p* = .051, see Table 2), while hit rates did not differ significantly (*p* > .05) between patients and controls. Median reaction times of patients were significantly slower than reaction times of controls (*p* = .001).

#### Alertness task

For the fMRI measurement of the alertness task, due to technical problems, reaction times were not available. Moreover, no errors were committed, therefore no statistical analyses were conducted for this task.

### Analysis of recruitment of functional networks in the three tasks

#### Comparison of components across the three cognitive tasks

**Fig 1:**
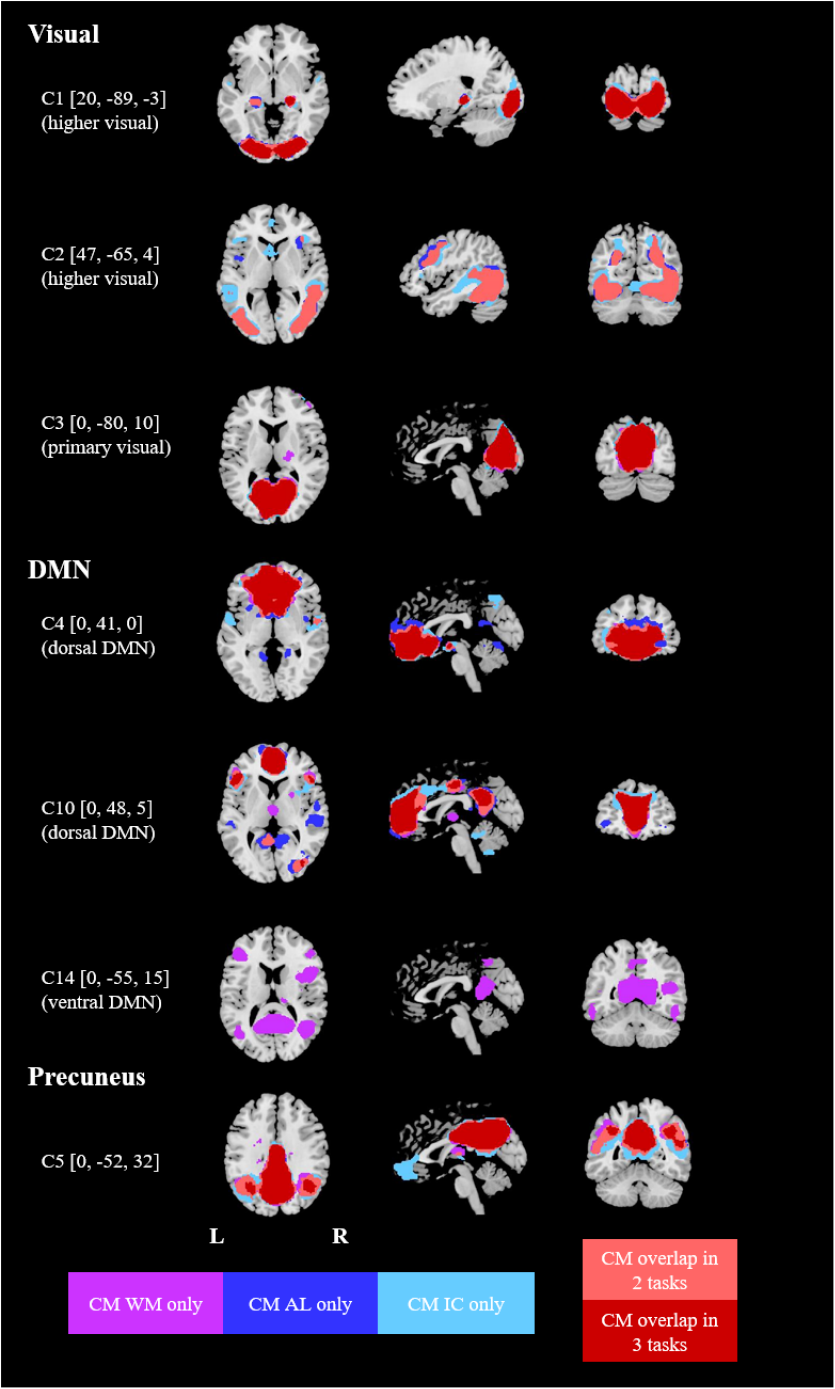
Spatially overlapping networks identified in the three cognitive tasks (part 1). MNI coordinates in brackets. CM = component map, WM = working memory task, AL = alertness task, IC = incompatibility task. CM overlap = areas which were included in the component maps of 2 [3] tasks.

**Fig 2:**
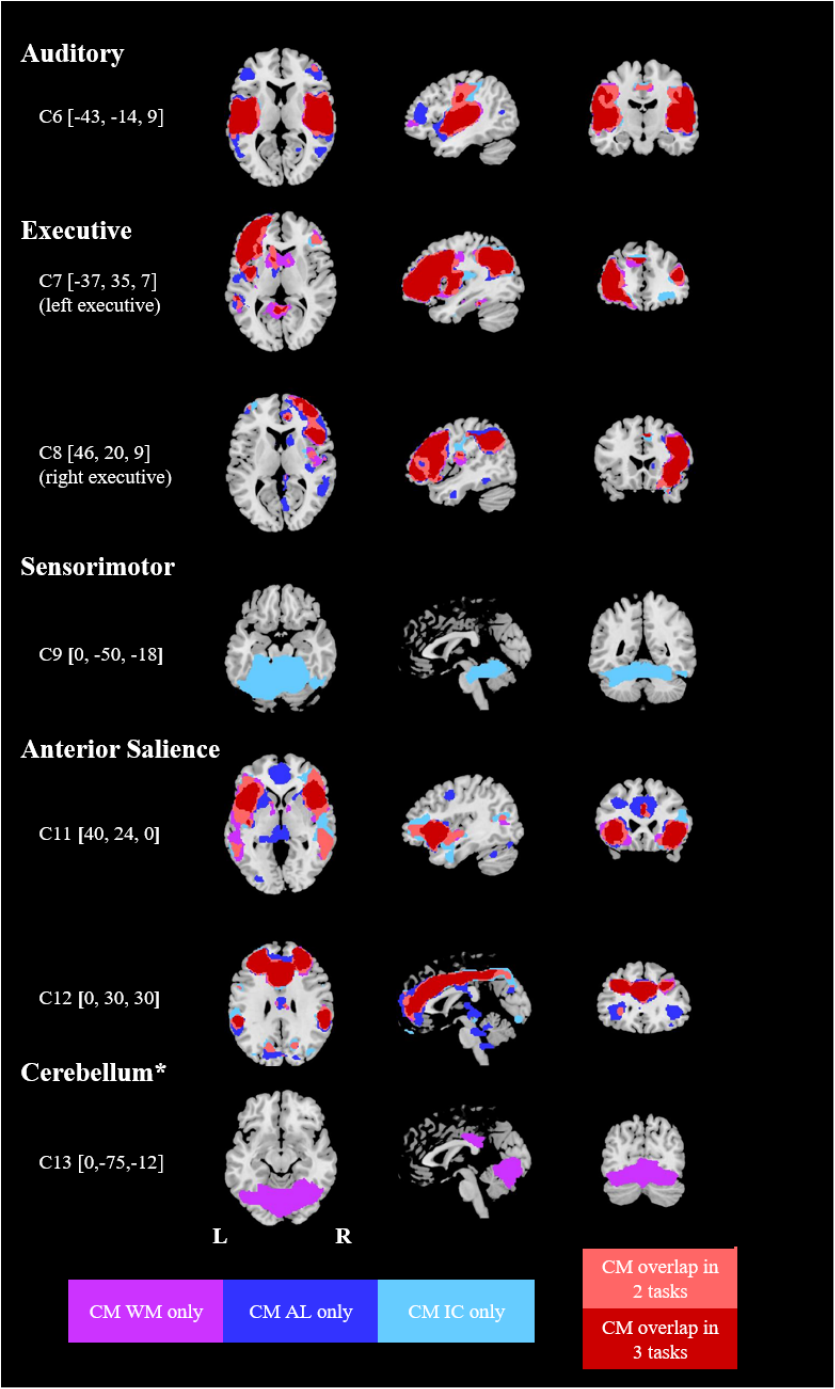
Spatially overlapping networks identified in the three cognitive tasks (part 2). MNI coordinates in brackets. CM = component map, WM = working memory task, AL = alertness task, IC = incompatibility task. CM overlap = areas which were included in the component maps of 2 [3] tasks. * = network comprising parts of the cerebellum, not identified in [39].

The component maps selected for each task showed a substantial spatial overlap between the three tasks (AL, IC, WM). Thus, 10 highly similar components could be visually matched across the three tasks (see Figs 1 and 2). Four additional components (C2, C9, C13, C14) were only identified in specific tasks (not in all three). By means of spatial overlaps, the component maps could be classified as parts of specific functional networks described in a previous study ([39], component maps downloadable at http://findlab.stanford.edu/functional_ROIs.html; see Figs 1 and 2). One component (C13) consisting of mainly cerebellar regions could not be matched and is included under the label “cerebellum”.

The task-modulation of each component is reflected in the beta weights shown in Table 3. Positive weights indicate an increased activity of the respective network during the active phase, while negative weights indicate a deactivation during the active task phase. In this section, we will first compare the components and their task-modulation in *healthy controls* across the three tasks before outlining differences between patients and controls in the following sections. As the focus of the present study was placed on cognitive functions, the results for visual, auditory and sensorimotor components will not be presented in detail here (but are included in Table 3). The spatial maps of all components are shown in Figs 1 and 2; their main brain regions are listed in Table 4.

**Table 3:** Beta weight values (*mean*(*SD*)) of the spatially matched components for each task.

	*IC*				*WM*				*AL*			
Component	Patients	Controls	*p* (2-sT)		Patients	Controls	*p* (2-sT)		Patients	Controls	*p* (2-sT)	
**Visual**												
C1	-1.17* (0.45)	-1.50* (0.33)	0.0347	C2	-0.86* (0.63)	-1.37* (0.37)	0.0215	C4	-1.21* (0.51)	-1.37* (0.39)	0.3775	C3
C2	0.53* (0.59)	0.81* (0.38)	0.1452	C3	n.i.	n.i.	n.i.		-0.05 (0.34)	0.36* (0.37)	0.0069**	C8
C3	-0.41* (0.32)	-0.22 (0.58)	0.3460	C4	-0.33 (0.45)	-0.01 (0.73)	0.2300	C3	-0.30* (0.35)	0.09 (0.63)	0.0727	C2
**DMN**												
C4	-0.63* (0.55)	-1.10* (0.44)	0.0178	C5	-0.30 (0.68)	-0.76* (0.42)	0.0520	C10	-0.44* (0.52)	-0.91* (0.37)	0.0098**	C1
C10	-0.67* (0.43)	-0.91* (0.35)	0.1036	C21	-1.08* (0.43)	-1.19* (0.40)	0.5210	C16	-0.60* (0.42)	-0.98* (0.26)	0.0056**	C20
C14	n.i.	n.i.	n.i.		-1.20* (0.24)	-1.04* (0.38)	0.2474	C28	n.i.	n.i.	n.i.	
**Precuneus**												
C5	-0.59* (0.27)	-0.71* (0.41)	0.3954	C7	-0.07 (0.44)	-0.11 (0.44)	0.8158	C15	-0.47* (0.35)	-0.52* (0.36)	0.7288	C16
**Auditory**												
C6	-0.38* (0.44)	0.10 (0.56)	0.0232	C9	-0.95* (0.31)	-0.64* (0.30)	0.0219	C18	0.05 (0.49)	0.46* (0.42)	0.0272	C6
**Executive**												
C7	0.28 (0.43)	0.46* (0.49)	0.3261	C10	1.10* (0.32)	1.10* (0.35)	0.9668	C17	-0.22 (0.37)	0.19 (0.41)	0.0131**	C10
C8	0.72* (0.35)	0.81* (0.42)	0.5720	C13	1.14* (0.24)	1.19* (0.22)	0.6221	C20	0.30 (0.46)	0.56* (0.40)	0.1307	C18
**Sensorimotor**												
C9	0.65* (0.35)	0.75* (0.44)	0.5511	C15	n.i.	n.i.	n.i.		n.i.	n.i.	n.i.	
**Anterior Salience**												
C11	-0.03 (0.37)	0.26* (0.40)	0.0717	C23	0.26 (0.54)	0.46* (0.35)	0.2910	C26	0.59* (0.39)	0.70* (0.46)	0.5365	C23
C12	0.38* (0.53)	0.83* (0.50)	0.0337	C24	0.47* (0.49)	0.82* (0.50)	0.0971	C23				
Cerebellum*												
C13	n.i.	n.i.	n.i.		0.21 (0.36)	0.57* (0.53)	0.0793	C19	n.i.	n.i.	n.i.	

IC = incompatibility task, WM = working memory task, AL = alertness task. p(2-sT) = p-value of 2 sample t-tests of beta weights between patients and controls. p-values are presented uncorrected and symbols “*/**” indicate significance at FDR-corrected thresholds as follows

* beta weight significant at p < .05 FDR-corrected in one-sample t-test (taking into account the number of components tested) and

** difference between beta weights significant at p < .05 FDR-corrected in 2-sample t-test.

Grey component numbers indicate the respective component number for each task (for reference in the respective 4D component maps). Underlined components contain clusters with min. 300 voxels classified as cerebellar. n.i. = not identified (respective component was not identified for this task).

**Table 4:** Main brain regions comprised in the component maps. Top brain regions included in the component maps as classified by AAL (automatic anatomic labelling) atlas (http://www.gin.cnrs.fr/AAL?lang=en) [36]. Labelling was conducted on binary masks consisting of the overlapping regions of the three tasks (component maps of each task FDR-thresholded at p_FDR_ < .001, *k* = 100). Please note that therefore no voxel intensity information is provided.

Comp	AAL regions	No of voxels	Comp	AAL regions	No of voxels
**C1**			**C8**		
	Occipital_Mid_L	505		Frontal_Mid_R	1310
	Lingual_R	440		Angular_R	948
	Occipital_Inf_L	392		Frontal_Inf_Oper_R	850
	Occipital_Inf_R	366		Frontal_Inf_Tri_R	790
	Lingual_L	332		Insula_R	503
**C2**			**C9**		
	Occipital_Mid_L	1582		Cerebelum_6_L	1282
	Fusiform_R	1539		Fusiform_L	1027
	Occipital_Mid_R	1016		Cerebelum_4_5_L	852
	Occipital_Sup_R	914		Fusiform_R	663
	Temporal_Mid_R	837		Cerebelum_4_5_R	660
				Cerebelum_6_R	599
**C3**			**C10**		
	Calcarine_L	1609		Frontal_Sup_Medial_L	1072
	Lingual_L	1428		Cingulum_Ant_L	775
	Calcarine_R	1399		Cingulum_Ant_R	744
	Lingual_R	1379		Frontal_Sup_Medial_R	637
	Cuneus_L	1101		Angular_L	527
	Cuneus_R	1026			
**C4**			**C11**		
	Rectus_L	696		Insula_L	885
	Rectus_R	652		Insula_R	800
	Frontal_Mid_Orb_R	611		Frontal_Inf_Orb_R	356
	Cingulum_Ant_L	601		Frontal_Inf_Orb_L	317
	Frontal_Sup_Orb_L	520		Frontal_Inf_Tri_L	316
	Frontal_Mid_Orb_L	515		Frontal_Inf_Tri_R	265
**C5**			**C12**		
	Precuneus_R	1540		Cingulum_Mid_R	1444
	Precuneus_L	1424		Cingulum_Mid_L	1297
	Cingulum_Mid_L	712		Frontal_Mid_L	1194
	Cingulum_Mid_R	687		Cingulum_Ant_L	926
	Angular_R	527		Frontal_Mid_R	650
	Cuneus_L	505		Insula_L	586
				SupraMarginal_R	560
**C6**			**C13**		
	Temporal_Sup_R	1550		Fusiform_R	1057
	Rolandic_Oper_R	1129		Cerebelum_6_R	1030
	Insula_R	931		Cerebelum_6_L	954
	Postcentral_R	679		Lingual_R	928
	SupraMarginal_R	417		Lingual_L	823
				Fusiform_L	783
				Cerebelum_Crus1_L	576
**C7**			**C14**		
	Frontal_Inf_Tri_L	1966		Precuneus_R	627
	Frontal_Mid_L	1331		Fusiform_R	591
	Parietal_Inf_L	1208		SupraMarginal_R	572
	Precentral_L	989		Calcarine_R	473
	Angular_L	868		Precuneus_L	465
	Frontal_Inf_Oper_L	765		Calcarine_L	445
	Insula_L	654		Frontal_Inf_Tri_L	423

The default mode network (DMN, represented in C4, C10 and C14) was deactivated during the active phase of all three tasks, as indicated by the negative beta-weights of the respective components (see Table 3). The precuneus (C5) also showed a negative task-modulation for IC and AL, but not for WM.

The left and right executive networks (C7 and C8) were positively task-modulated with the exception of C7 in AL. The anterior salience network (ASN, C11 and C12) was positively task-related in all three tasks. The cerebellar component C13 was only identified in the WM task and showed a positive task-modulation in controls.

#### Comparison of patients and controls

For the IC task, 12 components remained after the three-step component selection procedure (see section “MRI data analysis”). At an FDR-corrected level of significance, two-sample-t-tests of the beta-weights showed no significant differences between patients and controls (see Table 3). As described in the section “MRI data analysis”, for components containing cerebellar regions we additionally report results at p < .05 uncorrected for number of components tested. This was the case for the component maps of C4 (DMN) and C12 (anterior salience). The beta weights of both components indicated a stronger task modulation for controls than for patients (negative for C4 and positive for C12). Furthermore, there was a tendential correlation between beta-weights of C4 and reaction times (*r*_*s*_ = .31, *p* = .1), implicating that less deactivation of DMN was tendentially associated to slower reaction times. C12 beta-weights were not correlated with task performance.

For the WM task, 12 components remained after the three-step component selection procedure. Two-sample-t-tests of the beta-weights showed no significant differences between patients and controls in the components that were part of cognitive networks. As the WM task included a control condition (0-back task), additionally a mixed-measures ANOVA was conducted on beta-weights to assess whether task-modulation of components differed between the 2-back and 0-back tasks. The ANOVA yielded a significant main effect of task for 8 of the 12 selected components (*F*(1,22) = ranging from 6.2 to 82.7, *p* for all < .05 FDR-corrected): C1, C6, C7, C8, C10, C12, C13 and C14. For C1, C6, C10 and C14, a stronger negative task-modulation was observed for the 2-back compared to the 0-back task. For C7, C8, C12 and C13, a stronger positive task-modulation was observed in the 2-back task compared to the 0-back task.

Moreover, there was a significant main effect of group (*F*(1,22) = 10.1, *p*_*unc*_ = .004, *η*^2^ = .313) for C13, the cerebellar component. Interaction effects of task and group were not significant. Post-hoc tests of the group difference showed that the two group difference was mainly due to differential task-modulation in the 0-back-control task, not in the 2-back task: During the 0-back task, the time course of C13 was positively task-modulated in controls (*M* = .41, *SD* = .34), and showed no significant relation to the task in patients (*M* = -.098, *SD* = .27; *t*(22) = -3.88, *p* = .001). There was a significant negative correlation between working memory reaction times and beta weights of the 0-back task for C13 (*r*(22) = -.43, *p* = .036) while the correlation with beta weights of the 2-back task did not reach significance (*r*(22) = -.368, *p* = .077). Moreover, C13 beta-weights of the 2-back task were significantly related to accuracy in the 2-back task (*r*_*s*_ = .41, *p* = .049). All other correlations of task performance and beta weights did not reach significance (p > .1).

For the AL task, 10 components remained after the selection procedure. The task-relatedness of component time courses differed significantly between the two groups in C4, C7 and C10 (p < .05 FDR corrected, see Table 3): The time courses of C4 and C10 showed significantly more negative task-modulation in controls than in patients. C7 showed negative (though not significant) task-modulation in patients, and positive in controls.

### Analysis of task-related networks using GLM

To enable inclusion in meta-studies, we report activation differences in the three tasks emerging in a general linear model (GLM) analysis at a threshold of *p* < .001 uncorrected in S1 Text, but refrain from further interpretations of these activations in light of the potential unreliability of these effects [40].

## Discussion

The aim of the present study was to assess the long-term impact of cerebellar lesions after tumor treatment on cognitive functions and on global functional brain networks. Thus, during fMRI scanning, patients and healthy controls took part in three cognitive tasks covering an array of executive functions. Functionally connected networks during the tasks were identified by means of group independent component analysis (group ICA). Overall, several networks showed a decreased task-modulation in patients compared to controls, particularly in those tasks posing less cognitive demands. Fewer differences in functional networks were observed during cognitively more demanding tasks, possibly indicating a restoration of functional connectivity within networks involved in higher-order cognitive functions.

Overall cognitive functioning of all participants was evaluated by means of an IQ test. Patients differed significantly from healthy controls, as they showed lower standardized IQ scores than controls. However, comparisons with normative values showed that this difference was mainly due to the controls scoring higher than average, while patients’ scores did not differ from normative values. Thus, patients did not show a general cognitive deficit as reflected by IQ scores. Despite the controls’ higher IQ scores, the groups showed comparable performance in most parameters of the three tasks: The only significant difference observed was a slower reaction time in the working memory (WM) task for patients compared to controls. The comparable levels of task accuracy indicated patients’ general level of cognitive functioning was largely unaffected or restored, while slower reaction times might indicate a decreased processing speed, similar to the observations in a study on pediatric medulloblastoma survivors [41]. In general, these behavioral results concur with previous reports of rather mild cognitive deficits in patients with cerebellar lesions [42]. The finding that impairments were only visible in the domain of working memory are in line with previous evidence of a specific cerebellar involvement in this domain [42,43].

In line with previous studies applying ICA on task-based data (e.g. [39]), a number of cognitive networks implicated in task performance were identified in the present study: the default mode network (DMN), precuneus, the left and right executive network (LECN and RECN) and the anterior salience network (ASN).

For all three tasks the DMN was deactivated during the active task phase, which has been observed previously [44–46]. Moreover, the DMN was differentially modulated in the two groups: In the alertness (AL) and incompatibility (IC) tasks, patients showed a weaker deactivation of the dorsal DMN network as compared to controls. Precuneus was negatively task-modulated for IC and AL, but not for the higher-order WM task. This finding might implicate that this region is particularly involved in working memory processes (as it was not deactivated during the active phase as in the IC and AL tasks). These results are in line with a meta-analysis of studies investigating the n-back task, which identified precuneus as one amongst several areas consistently activated across all included n-back studies [47]. The comparable task-modulation in patients and controls indicates precuneus recruitment was not altered in patients.

The executive control networks were positively task-modulated, with controls showing an increased activity of LECN with higher cognitive load (WM > IC > AL). Interestingly, this was not the case for patients, as they showed no task-modulation of LECN in the IC task and an inversed task-modulation (i.e. negative beta-weight) in the AL task. The observed lack of task-modulation of LECN in patients might reflect subtle deficits in the recruitment of cognitive resources not strong enough to be reflected in task performance.

The ASN generally showed a positive task-modulation. In the IC task, a part of the ASN comprising particularly middle and anterior cingulate region, middle frontal gyrus and insula, showed a stronger positive task-modulation for controls than for patients. As these differences in task-modulation were not correlated to task-performance, they might be more related to structural/resting-state network differences between patients and controls. In line with this speculation, a recent study described altered resting-state connectivity in DMN, ECN and ASN [26] in survivors of childhood cerebellar tumors. The authors found an increased number of functional connections and stronger connections within these networks in survivors. One might speculate whether a hyperconnectivity in resting-state networks observed by Chen et al. [26] might lead to insufficient deactivation of these networks during task performance, as observed in the present study. Future studies focusing on the relation between resting-state and task-related networks might elucidate further how altered resting-state connectivity may impact task-modulation of functional networks.

In addition to the previously discussed networks, in the WM task a cerebellar component (C13) was identified. This network showed a stronger positive task-modulation in the 2-back task than in its control condition (0-back task). In line with previous studies [11,22–24], this result underscores the importance of the cerebellum for cognitive performance, specifically working memory. Previous studies have primarily associated superior/lateral cerebellar regions (lobule VI, Crus1) with articulatory control and posterior/inferior cerebellar regions with phonological storage and monitoring [48–50]. The cerebellar component identified in the present study comprised mainly the left lobule VI and Crus 1 of the cerebellar hemisphere, suggesting a potential role of this component in internal rehearsal of the numbers presented in the WM task. Group differences were observed mainly in the 0-back task, emerging from a stronger positive task-modulation of this component in controls compared to patients. These results might indicate that the functional connectivity of the cerebellum was not entirely restored in patients. There is ample evidence for the existence of cerebro-cerebellar loops involved in cognitive functioning. In particular, high cognitive demand was related to cerebellar connections to prefrontal and parietal cortices [51,52]. In line with these findings, in the present study the cerebellum was part of default mode and anterior salience components. The respective default mode network (C4) comprised mainly parts of prefrontal cortex, while the respective anterior salience network (C12) contained cingular, insular and frontal regions, pointing to widespread connections of the cerebellum during this task. These components showed a weaker task-modulation in patients in the IC task, possibly indicating an incomplete restoration of the networks in patients. Of note, as patients did not show impaired IC task performance, our results suggest that either the deficient network recruitment was compensated by the use of differential strategies in patients or these networks are not essential for task completion.

Overall, contrary to our predictions, most group differences in functional connectivity were observed in the least cognitively demanding tasks (AL and the WM 0-back control condition, which is highly similar to AL). Meanwhile, only slight differences were visible in the higher-order cognitive tasks IC and WM. Particularly, an incomplete deactivation of the DMN and a weaker task-modulation of the ECN was observed for the AL task in patients. These basic differences might have been “overshadowed” by the activity of higher-order networks in the two other tasks. The comparable task-modulation of networks for the IC and WM tasks might suggest that a partial reorganization of the networks required for execution of these higher-order tasks occurred in patients. Of note, while functional reorganization can explain the result patterns we observed in patients with longstanding lesions, this might not be the case in patients with progressive degenerative cerebellar diseases [53–55].

## Limitations

Although our sample was very homogenous regarding tumor type, tumor location, time between surgery and experiment, and age-matching of healthy controls, there was a large time gap (*mean* 56 days) between the neuropsychological testing and MRI measurements. Although spontaneous neuronal reorganization during this time is very unlikely, as the surgery of our patients had been conducted long before (7–20 years), we cannot exclude other possible confounds arising from this gap. Moreover, the small sample size resulting from the selection of a homogeneous patient group may be a limiting factor in the interpretation of our results.

Furthermore, the results of our cognitive assessment indicated that the control group scored higher than average in the IQ test. This difference was a result of the fact that our control subjects were medical students. Subject matching procedure was based on age and gender and did not include level of education and socioeconomic status. Despite the above-average cognitive ability of the control group, patients and controls showed little differences in performance in the three specific tasks performed in our study. Therefore, it is unlikely that the connectivity differences we observed emerge from the over-achieving control group, rather than from effects of the cerebellar lesions in our patients. Still, future studies should consider IQ during control group acquisition.

## Conclusion

In summary, patients in our study showed no impairments in general cognitive functioning (as reflected by IQ scores) and only slightly impaired performance in the three specific cognitive tasks (working memory, incompatibility, and alertness) conducted.

Largest differences in the recruitment of functional networks between patients and controls were observed in the most basic task (alertness) employed in the present study, while fewer differences were observed in the higher-order tasks (incompatibity and working memory). Thus, our results might reflect a partial restoration of the synchronicity of brain activity (as reflected by functional network connectivity) in patients in the course of neuroplastic reorganization, particularly for networks related to higher-order cognitive processes.

While the block design implemented in the present study allowed us the study of general state-related processes during the cognitive tasks, future studies implementing event-related designs will enable a more fine-grained analysis allowing the disentanglement of trials related to error processing and correct task responses. Moreover, as our aim was the investigation of global functional networks, we used independent component analysis, which represents a non-targeted approach regarding predefined regions. Future studies employing more directed functional connectivity approaches or comparing the effect of different lesion locations within the cerebellum will shed more light on the role of cerebellar substructures for cognitive functioning.

The complex activation patterns observed in our study underline the importance of testing several cognitive functions to assess the specificity of cognitive deficits and neuronal reorganization processes after brain lesions.

## Supporting information

S1 Fig Procedure of the AL (alertness) task. The task was presented in block design including 6 baseline and 5 active blocks (30 seconds per block) with an overall duration of five and a half minutes. During active blocks, targets (crosses) appeared at randomly varying intervals (ISI 2100–2300 ms) in the middle of the screen. Subjects were instructed to respond as quickly as possible by button press (right index finger) every time a cross appeared. Each active block contained 9–12 targets (stimulus duration max. 2 seconds, response-terminated). As the target presentation was response-terminated, the number of targets in each (30-second) block varied depending on the subject’s reaction time. During the baseline periods, subjects were presented with a black screen displaying hash symbols.
(TIF)

S2 Fig Procedure of the IC (incompatibility) task. The task was presented in block design including 6 baseline and 5 active blocks (30 seconds per block) with an overall duration of five and a half minutes. During active blocks, arrows pointing either to the left or the right were presented on the left or the right side of a fixation cross located in the middle of the screen. Subjects were instructed to respond by either a left or right button press depending on the direction the arrow was pointing, irrespective of the side on which the arrow was appearing (i.e. left index finger button press for an arrow pointing to the left, even if it was presented on the right side). The arrows were counter-balanced regarding side of presentation and direction they were pointing (left/right). Each trial started with a warning signal (cross) that was presented for 1 s on the screen. Subsequently, the arrow appeared (stimulus duration 1s). The ISI between arrow and next warning signal was 600 ms. Active blocks contained 11–12 arrows. During the baseline periods of the stimuli design, subjects were presented with a black screen displaying hash symbols.
(TIF)

S3 Fig Procedure of the WM (working memory) task. The task was presented in block design including 6 baseline and 5 active blocks (30 seconds per block) with an overall duration of five and a half minutes. During the working memory (2-back) task, a randomized sequence of one-digit numbers was presented on the screen. During each active block, 7 numbers (stimulus duration 1500 ms) were presented with an ISI of 3000 ms between numbers. Subjects were required to determine whether the number currently shown corresponded with the last but one number. The active blocks contained a total of 2–7 targets (i.e. numbers that were the same as the last but one number). During the baseline periods of the stimuli design, subjects were presented with a black screen displaying hash symbols.
(TIF)

S4 Fig Procedure of the 0-back (working memory control) task. The 0-back task was presented in block design, including 6 baseline and 5 active blocks (30 seconds per block) with an overall duration of five and a half minutes. The subjects were instructed to press a button every time a pre-defined number (stimulus duration 1500 ms) was presented. Each active block contained 7 numbers, and active blocks contained a total of 2–7 targets. During the baseline periods of the stimuli design, subjects were presented with a black screen displaying hash symbols.
(TIF)

S1 Table Remaining number of components after each preselection step. (DOCX)

S2 Table Detailed description of task parameters employed in the MRI setting. (DOCX)

S1 Text Analysis of task-related networks using GLM. (DOCX)

S1 Dataset Behavioral and demographic data of patients and controls. The data set contains the subject code, age, sex, group (0 = patient, 1 = control), IQ, time since surgery (OP_fMRI) and task accuracy and reaction times for the IC and WM tasks.
(CSV)

S2 Dataset Component beta-weights of the IC task. (CSV)

S3 Dataset Component beta-weights of the WM task and 0-back control task. (CSV)

S4 Dataset Component beta-weights of the AL task. (CSV)

S5 Dataset Component time courses of the IC task (subject order as in S1 Dataset, column ‘subj_code_al_ic’). (ZIP)

S6 Dataset Component time courses of the WM task and 0-back control task (subject order as in S1 Dataset, column ‘subj_code_wm’). (ZIP)

S7 Dataset Component time courses of the AL task (subject order as in S1 Dataset, column ‘subj_code_al_ic’). (ZIP)

S1 File GLM T-map (NIFTI format) for patients > controls for the IC task (see S1 Text). (NII)

S2 File GLM T-map (NIFTI format) for controls > patients for the IC task (see S1 Text). (NII)

S3 File GLM T-map (NIFTI format) for patients > controls for the WM task (see S1 Text). (NII)

S4 File GLM T-map (NIFTI format) for controls > patients for the WM task (see S1 Text). (NII)

S5 File GLM T-map (NIFTI format) for patients > controls for the AL task (see S1 Text). (NII)

S6 File GLM T-map (NIFTI format) for controls > patients for the AL task (see S1 Text). (NII)
