# Supplementary figures and images for: Neuronal correlates of cognitive function in patients with childhood cerebellar tumor lesions
Source: PLoS One. 2017 Jul 10;12(7):e0180200. doi: 10.1371/journal.pone.0180200 (PMC5503240; doi:10.1371/journal.pone.0180200)

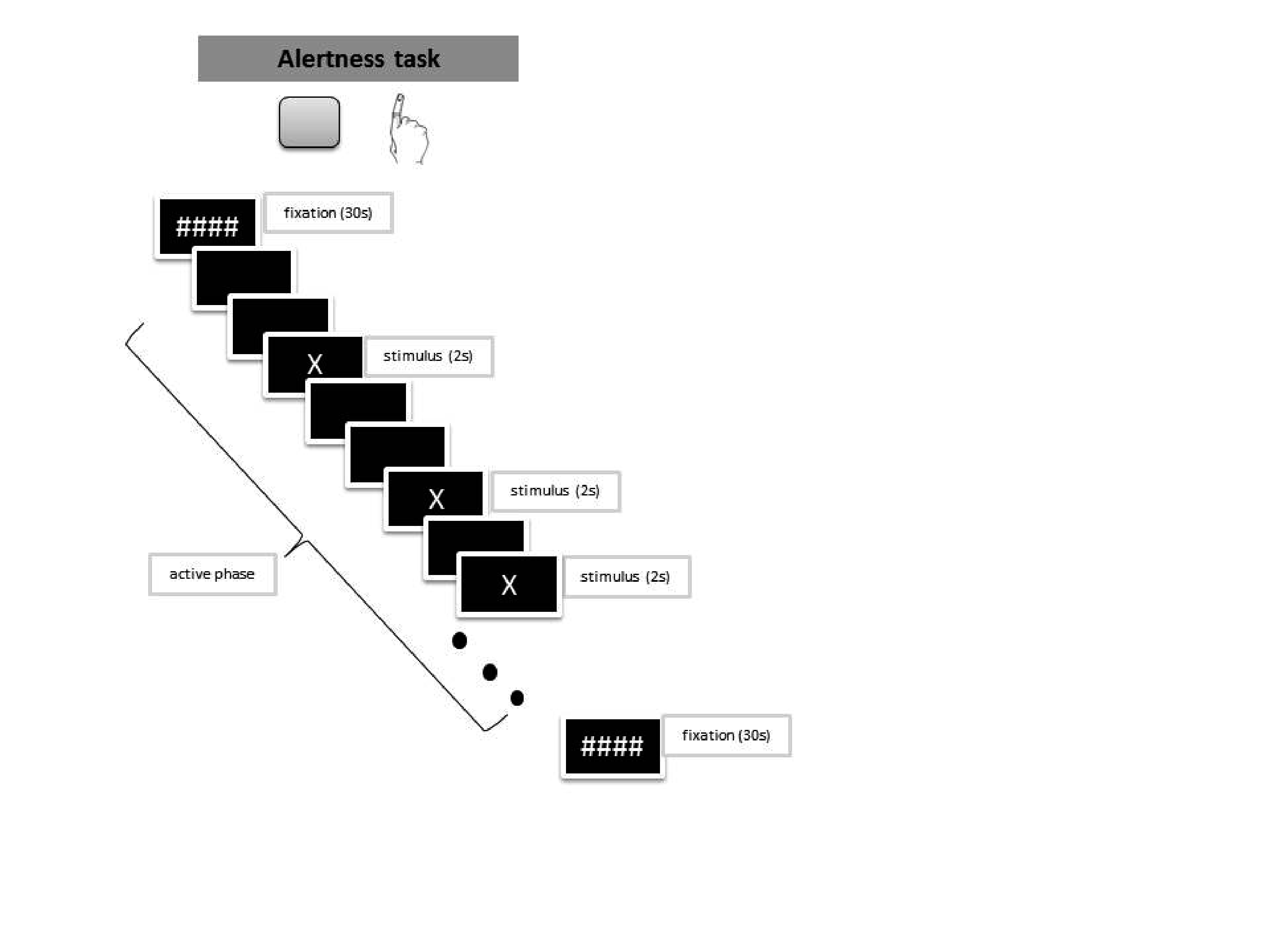

Supplement: S1 Fig — The task was presented in block design including 6 baseline and 5 active blocks (30 seconds per block) with an overall duration of five and a half minutes. During active blocks, targets (crosses) appeared at randomly varying intervals (ISI 2100–2300 ms) in the middle of the screen. Subjects were instructed to respond as quickly as possible by button press (right index finger) every time a cross appeared. Each active block contained 9–12 targets (stimulus duration max. 2 seconds, response-terminated). As the target presentation was response-terminated, the number of targets in each (30-second) block varied depending on the subject’s reaction time. During the baseline periods, subjects were presented with a black screen displaying hash symbols. (TIF) [file pone.0180200.s001.tif]

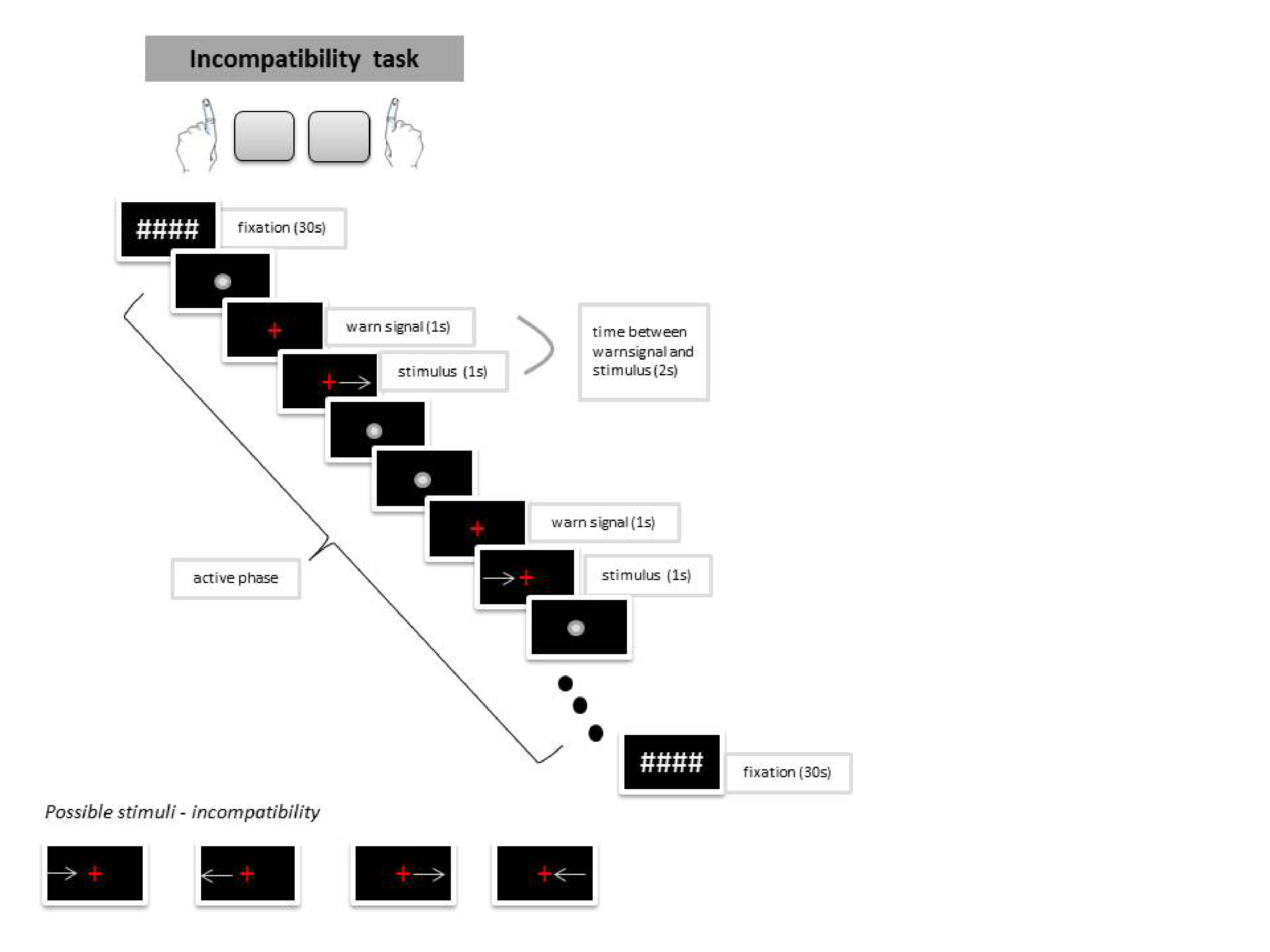

Supplement: S2 Fig — The task was presented in block design including 6 baseline and 5 active blocks (30 seconds per block) with an overall duration of five and a half minutes. During active blocks, arrows pointing either to the left or the right were presented on the left or the right side of a fixation cross located in the middle of the screen. Subjects were instructed to respond by either a left or right button press depending on the direction the arrow was pointing, irrespective of the side on which the arrow was appearing (i.e. left index finger button press for an arrow pointing to the left, even if it was presented on the right side). The arrows were counter-balanced regarding side of presentation and direction they were pointing (left/right). Each trial started with a warning signal (cross) that was presented for 1 s on the screen. Subsequently, the arrow appeared (stimulus duration 1s). The ISI between arrow and next warning signal was 600 ms. Active blocks contained 11–12 arrows. During the baseline periods of the stimuli design, subjects were presented with a black screen displaying hash symbols. (TIF) [file pone.0180200.s002.tif]

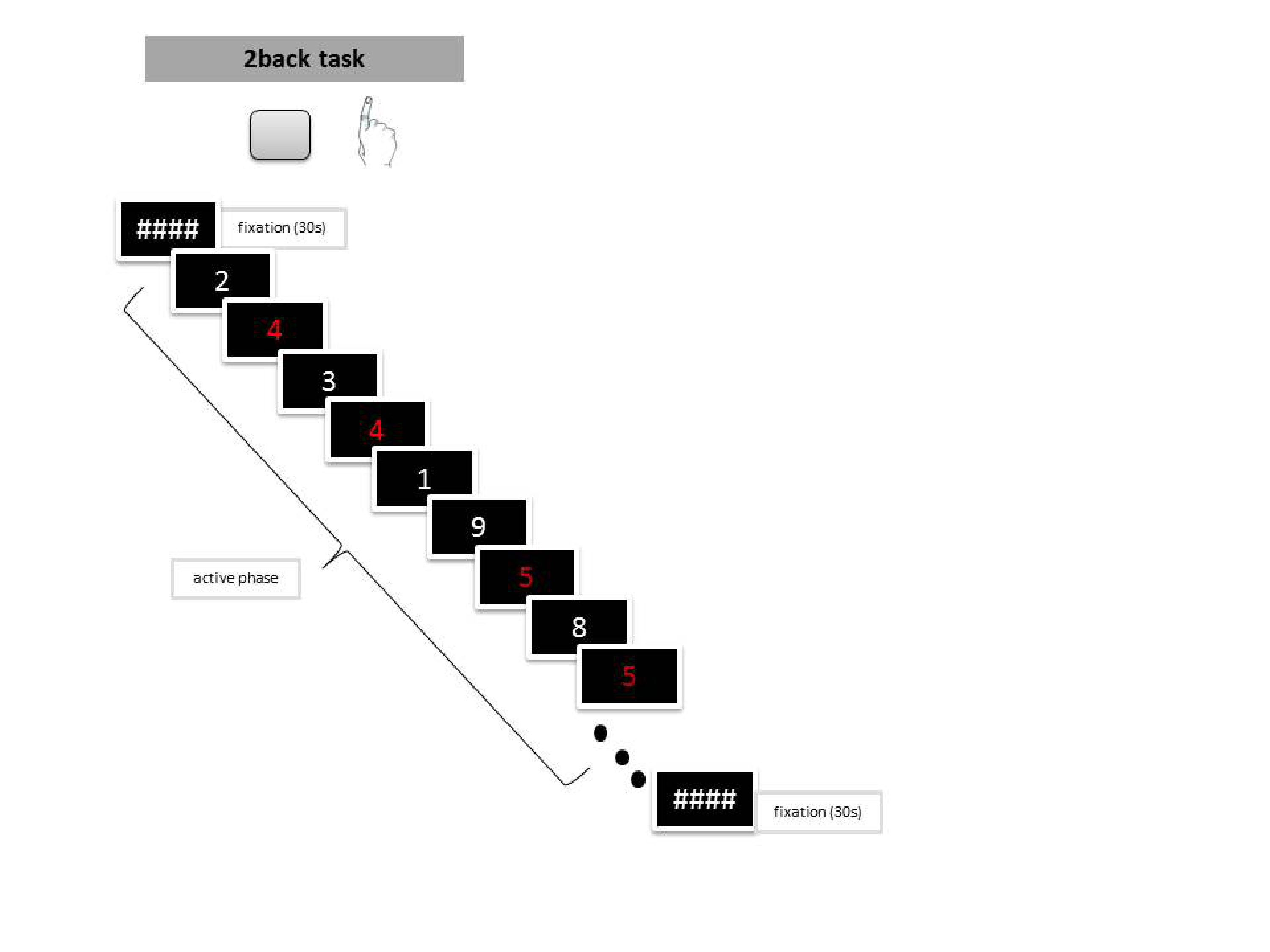

Supplement: S3 Fig — The task was presented in block design including 6 baseline and 5 active blocks (30 seconds per block) with an overall duration of five and a half minutes. During the working memory (2-back) task, a randomized sequence of one-digit numbers was presented on the screen. During each active block, 7 numbers (stimulus duration 1500 ms) were presented with an ISI of 3000 ms between numbers. Subjects were required to determine whether the number currently shown corresponded with the last but one number. The active blocks contained a total of 2–7 targets (i.e. numbers that were the same as the last but one number). During the baseline periods of the stimuli design, subjects were presented with a black screen displaying hash symbols. (TIF) [file pone.0180200.s003.tif]

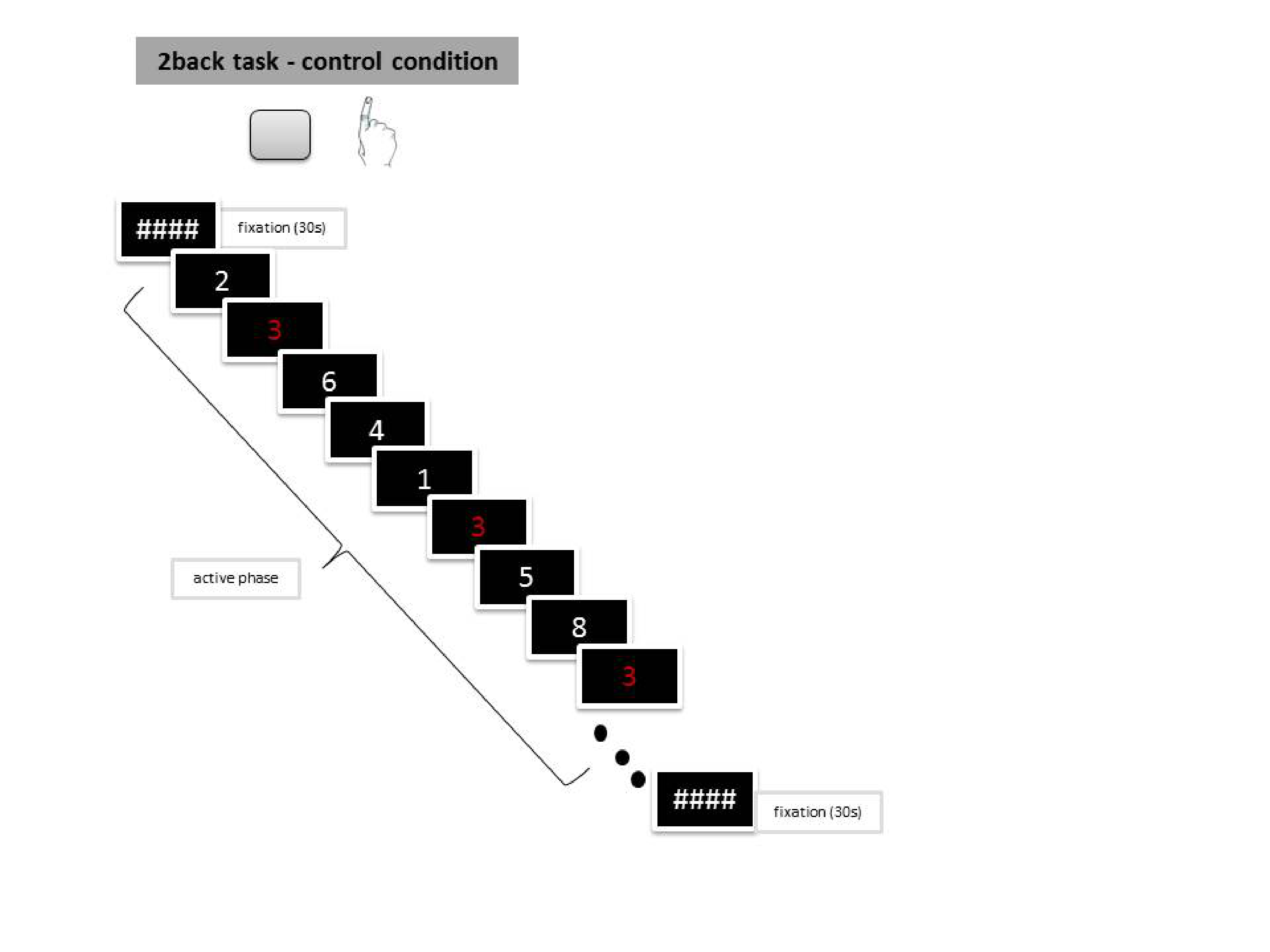

Supplement: S4 Fig — The 0-back task was presented in block design, including 6 baseline and 5 active blocks (30 seconds per block) with an overall duration of five and a half minutes. The subjects were instructed to press a button every time a pre-defined number (stimulus duration 1500 ms) was presented. Each active block contained 7 numbers, and active blocks contained a total of 2–7 targets. During the baseline periods of the stimuli design, subjects were presented with a black screen displaying hash symbols. (TIF) [file pone.0180200.s004.tif]
