# Supplementary material for: Neuronal correlates of cognitive function in patients with childhood cerebellar tumor lesions
Source: PLoS One. 2017 Jul 10;12(7):e0180200. doi: 10.1371/journal.pone.0180200 (PMC5503240; doi:10.1371/journal.pone.0180200)
Supplement: S1 Table — (DOCX) [file pone.0180200.s005.docx]

S1 Table. Remaining number of components after each preselection step.

|  | Total estimated no of components | Remaining after 1st step | Remaining after 2^nd^ step | Remaining after 3^rd^ step |
| --- | --- | --- | --- | --- |
| Incompatibility task | 28 | 19 | 15 | 12 |
| Working memory task | 31 | 20 | 14 | 12 |
| Alertness task | 29 | 18 | 12 | 10 |
