# Supplementary material for: Neuronal correlates of cognitive function in patients with childhood cerebellar tumor lesions
Source: PLoS One. 2017 Jul 10;12(7):e0180200. doi: 10.1371/journal.pone.0180200 (PMC5503240; doi:10.1371/journal.pone.0180200)
Supplement: S2 Table — (DOCX) [file pone.0180200.s006.docx]

S2 Table. Detailed description of task parameters employed in the MRI setting.

| **Alertness** |  |  |
| --- | --- | --- |
|  | total no. of targets | 45-60 (dependent on RT) |
|  | ISI | random variation between 2100 – 2300 ms |
|  | target stimulus duration | max. 2000 ms (response-terminated) |
|  | total task duration | 5 ½ minutes |
| **Incompatibility** |  |  |
|  | total no. of stimuli | 58 |
|  | target stimulus duration | 1000 ms warning signal (cross), 1000 ms target |
|  | ISI | 600 ms |
|  | total task duration | 5 ½ minutes |
| **Working memory** |  |  |
|  | total no. of stimuli | 35 |
|  | total no. of targets (% of stimuli) | 2-7 (5-20%) |
|  | stimulus duration | 1500 ms |
|  | ISI | 3000 ms |
|  | total task duration | 5 ½ minutes |
