# Supplementary material for: Neuronal correlates of cognitive function in patients with childhood cerebellar tumor lesions
Source: PLoS One. 2017 Jul 10;12(7):e0180200. doi: 10.1371/journal.pone.0180200 (PMC5503240; doi:10.1371/journal.pone.0180200)
Supplement: S1 Text — (DOCX) [file pone.0180200.s007.docx]

**GLM analysis**

First level analysis was performed for each paradigm separately. Statistical parametric maps of the *t*-statistic for active versus baseline (active > baseline) contrasts were constructed for each subject for the alertness and the incompatibility task. In order to test for relative effects of the working memory task compared to the continuous vigilance performance of the 0-back task, the contrast 2-back (active-baseline) > 0-back (active-baseline) was used.

Corresponding contrast images were entered into a second level analysis, performing a two sample *t*-test for each paradigm respectively, in order to delineate differences in brain activations between healthy controls and patients after cerebellar tumor surgery. For second level analysis, two contrasts were created: patients > controls; controls > patients.

Results at an uncorrected statistical threshold of p < .001 and with a minimum cluster extent of k = 10 are shown in Tables A –C.

Table A. Significantly higher activated brain areas for the two contrasts patients > controls and controls > patients in the incompatibility task

| **patients > controls** | |  | MNI coordinates | | |
| --- | --- | --- | --- | --- | --- |
| Cluster size^a^ | Anatomical label^b^ | p-value^c^ | X | Y | Z |
| 99 | Right middle temporal pole | < .001 | 36 | 10 | -32 |
| 99 | Left inferior temporal gyrus | < .001 | -36 | -4 | -32 |
| 81 | Left anterior cingulate gyrus | < .001 | -10 | 34 | 0 |
| 75 | Right gyrus rectus | < .001 | 12 | 30 | -14 |
| 52 | Left gyrus rectus | < .001 | -6 | 32 | -16 |
|  |  |  |  |  |  |
| **controls > patients** | |  |  | | |
| 27 | Left inferior frontal gyrus, pars opercularis | < .001 | -54 | 8 | 20 |
| 12 | Left insula | < .001 | -32 | 24 | 2 |

^a^ Significantly activated clusters with more than 10 voxels

^b^ Clusters were automatically labeled using AAL Atlas [1].

^c^ *p* <.001 uncorrected

Table B. Significantly higher activated brain areas in controls compared to patients regarding the alertness task.

| **controls > patients** | |  | MNI coordinates | | |
| --- | --- | --- | --- | --- | --- |
| Cluster size^a^ | Anatomical label^b^ | p-value^c^ | X | Y | Z |
| 439 | **Left inferior frontal gyrus** | < .001 | -52 | 16 | 22 |
| 330 | **Lobule VI of vermis** | < .001 | -4 | -72 | -14 |
| 218 | Lobule VIII of vermis | < .001 | 6 | -70 | -36 |
| 183 | Left inferior temporal gyrus | < .001 | -56 | -52 | -6 |
| 74 | Left superior temporal gyrus | < .001 | -50 | -44 | 16 |
| 29 | Left postcentral gyrus | < .001 | -60 | -18 | 30 |
| 16 | Right superior temporal gyrus | < .001 | 64 | -34 | 8 |

^a^ Significantly activated clusters with more than 10 voxels

^b^ Clusters were automatically labeled using AAL atlas [1].

^c^ p < .001 uncorrected. Bold clusters were significant at p <.05 cluster-level FWE correction

Table C. Significantly higher activated brain areas in controls compared to patients regarding the working memory task.

| **patients > controls** | |  | MNI coordinates | | |
| --- | --- | --- | --- | --- | --- |
| Cluster size^a^ | Anatomical label^b^ | p-value^c^ | X | Y | Z |
| 65 | Right precuneus, right posterior cingulum, right lingual gyrus | < .001 | 8 | -34 | 8 |
| 11 | Left posterior cingulum | .001 | -6 | -38 | 6 |
| **controls > patients** | |  |  | | |
| 20 | Middle temporal gyrus | < .001 | 56 | -30 | -4 |
|  |  |  |  |  |  |

^a^ Significantly activated clusters with more than 10 voxels

^b^ Clusters were automatically labeled using AAL atlas [1].

^c^ p < .001 uncorrected. Bold clusters were significant at p <.05 cluster-level FWE correction

References

1. Tzourio-Mazoyer N, Landeau B, Papathansassiou D, Crivello F, Etard O, Delcroix N, et al. Automated anatomical labeling of activations in SPM using a macroscopic anatomical parcellation of the MNI MRI single-subject brain. Neuroimage. 2002;15: 273–289.
